# Comparative Proteomic Analysis of Embryos between a Maize Hybrid and Its Parental Lines during Early Stages of Seed Germination

**DOI:** 10.1371/journal.pone.0065867

**Published:** 2013-06-11

**Authors:** Baojian Guo, Yanhong Chen, Guiping Zhang, Jiewen Xing, Zhaorong Hu, Wanjun Feng, Yingyin Yao, Huiru Peng, Jinkun Du, Yirong Zhang, Zhongfu Ni, Qixin Sun

**Affiliations:** 1 State Key Laboratory for Agrobiotechnology and Key Laboratory of Crop Heterosis and Utilization (MOE), Beijing Key Laboratory of Crop Genetic Improvement, China Agricultural University, Beijing, China; 2 National Plant Gene Research Centre (Beijing), Beijing, China; New Mexico State University, United States of America

## Abstract

In spite of commercial use of heterosis in agriculture, the molecular basis of heterosis is poorly understood. It was observed that maize hybrid Zong3/87-1 exhibited an earlier onset or heterosis in radicle emergence. To get insights into the underlying mechanism of heterosis in radicle emergence, differential proteomic analysis between hybrid and its parental lines was performed. In total, the number of differentially expressed protein spots between hybrid and its parental lines in dry and 24 h imbibed seed embryos were 134 and 191, respectively, among which 47.01% (63/134) and 34.55% (66/191) protein spots displayed nonadditively expressed pattern. Remarkably, 54.55% of nonadditively accumulated proteins in 24 h imbibed seed embryos displayed above or equal to the level of the higher parent patterns. Moreover, 155 differentially expressed protein spots were identified, which were grouped into eight functional classes, including transcription & translation, energy & metabolism, signal transduction, disease & defense, storage protein, transposable element, cell growth & division and unclassified proteins. In addition, one of the upregulated proteins in F_1_ hybrids was ZmACT2, a homolog of *Arabidopsis thaliana* ACT7 (AtACT7). Expressing *ZmACT2* driven by the *AtACT7* promoter partially complemented the low germination phenotype in the *Atact7* mutant. These results indicated that hybridization between two parental lines can cause changes in the expression of a variety of proteins, and it is concluded that the altered pattern of gene expression at translational level in the hybrid may be responsible for the observed heterosis.

## Introduction

Heterosis or hybrid vigor was defined as the advantage of hybrid performance over its parents in terms of biomass, size, yield, speeds of development, fertility, resistance to biotic and abiotic stresses. However, the genetic and molecular basis of heterosis remains enigmatic [1]. At the genomic level, a significant loss of colinearity at many loci between different inbred lines of maize was observed [2]. At the level of gene expression, complex transcriptional networks specific for different developmental stages and tissues in maize, wheat, rice and *Arabidopsis* were monitored, and these results indicated that hybridization between two parental lines could cause expression changes of different genes, which might be responsible for the observed heterosis [3]–[10]. Although transcriptomic analysis have contributed greatly to our understanding of the heterosis, changes on the level of mRNA do not necessarily indicate changes on the protein level, thus studies are needed to determine the differential proteomes between hybrids and its parental lines, and understand their functional roles in heterosis.

In fact, as early as 1970s, several investigators had estimated the correlations between isozyme allelic diversity and grain yield of single-cross maize hybrid [11]–[13]. Lately, two-dimensional gel electrophoresis was employed to determine correlations between polymorphism of individual protein amounts indices and hybrid vigor for agronomic traits [14]–[18]. For example, comparative proteomic analysis of 25 and 35 DAP (days after pollination) seed embryos of maize reciprocal F_1_-hybrids and their parental inbred lines revealed that 141 proteins exhibited nonadditive accumulation in at least one hybrid and approximately 44% of differently expressed proteins displayed low-dominant in hybrid [17]. Taken together, these observations at translational level add circumstantial evidence that expression differences between hybrid and its parental lines exist not only at mRNA levels, but also at protein abundances.

In maize, F_1_ hybrid seeds have a superior germination capacity as compared to their parental inbred lines, and hybrid vigor in maize is detectable at early stages of germination [19]. Studies have also indicated that vigorous growth of the embryonic axis in germinating F_1_ seed is related to a higher rate of RNA and protein synthesis [20]. Recently, proteomic analysis of heterosis during maize seed germination was analyzed and 257, 363, 351, 242, and 244 nonadditively expressed proteins were identified in hybrids Zhengdan 958, Nongda 108, Yuyu 22, Xundan 20, and Xundan 18 corresponding to their parents, respectively. Additionally, 54 different proteins were identified and the most interesting were those involved in germination-related hormone signal transduction, including abscisic acid and gibberellin regulation networks [21]. In the present study, high-throughput two-dimensional gel electrophoresis (2-DE) was used to establish the differentially expressed protein profiles in dry and 24 h imbibed embryos of maize hybrid Zong3/87-1 and its parental lines and differentially expressed proteins were further identified by MALDI TOF MS, with the purpose to gain insights into the molecular basis of maize heterosis of radicle emergence.

## Materials and Methods

### Plant Materials and Total Protein Extraction from Embryo

One highly heterotic hybrid Zong3/87-1 and its female parent Zong3 and male parent 87-1 were selected for this study. Germination efficiency was determined in three replicates. For each replicate, 100 seeds were placed embryo side down on two pieces of Whatman Grade No. 1 filter paper and placed in a plastic Petri dish, which were incubated at 28°C in 24 h dark. Seeds were considered germinated when radicle protrusion was visible. We recorded the germination rate at 12, 24, 36, 48 and 60 h after imbibitions in terms of radicle emergence, and the changes of 100 seeds weight were recorded during the germination process. Statistical analysis of the differences in traits was performed by using *t*-test.

The dry and 24 h imbibed embryos were detached from seeds, respectively. Remarkably, since germination of a batch of maize seeds was not strictly synchronous, 24 h imbibed embryos of each genotype with no radicle emergence were dissected and mixed for protein extraction and three replicates were performed. Twenty embryos were pooled as one biological replicate for protein extraction, and then frozen in liquid nitrogen stored at -80°C before use. Total protein was isolated from embryos using Invitrogen’s TRIZOL® reagent according to the manufacturer’s instruction. Protein concentration was determined by Ramagli assay [22].

### 2-DE and Image Analysis

The seed embryo proteins in the dried powder were solubilized in 7 M urea, 2 M thiourea, 2% CHAPS (powder to solution, w/v), 0.5% IPG buffer (v/v) (pH 4–7) (GE Healthcare, USA) and 36 mM DTT (5.6 mg/mL) via incubation at room temperature for 1 h, vortexing every 10 min, the mixture was then centrifuged (15 000 rpm) for 15 min, and the supernatant was collected. Total protein extract (500 µg) was loaded onto GE Healthcare 24 cm IPG gel strips (pH 4–7) during strip rehydration overnight. IEF of the acidic range IPG strips (pH 4–7) were conducted using IPGPhor II (GE Healthcare, USA) at 20°C for a total of 65 kVh. The IPG strips were equilibrated according to the manufacturer (GE Healthcare, USA). The second dimension SDS-PAGE gels (12.5% linear gradient) were run on an Ettan Daltsix (GE Healthcare, USA), 0.5 h at 2.5 W per gel, then at 15 W per gel until the dye front reached the gel bottom. Upon electrophoresis, the protein spots were stained with silver nitrate according to the instruction of protein PlusOne™ Silver Staining Kit (GE Healthcare, USA), which offered improved compatibility with subsequent mass spectrometric analysis. Briefly, gels were fixed in 40% ethanol and 10% acetic acid for 30 min, and then sensitized with 30% ethanol, 0.2% sodium thiosulfate (w/v) and 6.8% sodium acetate (w/v) for 30 min. Then gels were rinsed with distilled water three times, five minutes for each time, then incubated in silver nitrate (2.5 g/L) for 20 min. Incubated gels were rinsed with distilled water and developed in a solution of sodium carbonate (25 g/L) with formaldehyde (37%, w/v) added (300 µL/L) before use. Development was stopped with 1.46% EDTA-Na_2_·2H_2_O (w/v), and gels were stored in distilled water until they could be processed and the reproducible spots removed from them. Gel images were acquired using Labscan (GE Healthcare, USA) at 300 dpi resolution. Image was analyzed with Imagemaster 2D Platinum Software Version 5.0 (GE Healthcare, USA). Spot detection was performed with the parameters smooth, minimum area and saliency set to 2, 15 and 8, respectively, and was done automated by the software used, followed by manual spot editing, such as artificial spot deletion, spot splitting and merging. The experiments were carried out in triplicate. Only those protein spots that could be detected in all three replicated gels were considered for further analysis.

### Protein Expression Patterns Analysis

All the gels were matched to the reference gel in automated mode, combined with manual pair correction. The volume of each spot from three replicate gels was normalized and quantified against total spot volume. In this analysis the parental lines midparent value (the average value of the parental inbred lines) was calculated for all three replicates and compared with the hybrid expression for each of the three replicates. Only those protein spots with the fold changes more than 1.5 and significant at *p*<0.05 were considered as differentially expressed protein between hybrid and its parental lines. Among which any deviation from the midparent value was considered nonadditive expressed proteins, others were consider as additive protein. Accordining to the system suggested by Hoecker et al., the nonadditive proteins were further grouped into the following possible modes [16], including (i) above high parent expression (++), the expression in the hybrid significantly exceeded both parental inbred lines; (ii) below low parent expression (–), the expression in the hybrid was significantly lower than in both parents; (iii) high parent expression (+), expression in hybrid is equal to the highly expressed parent, but significantly difference from low expressed parent; (iv) low parent (-), expression in hybrid is equal to the lowly expressed parent, but significantly distinct from the highly expressed parent; (v) partial dominance (+/−), the expression abundance of the hybrid protein is significantly higher than the lower performing parent and significantly lower than the higher performing parent; (vi) different (D), displayed an expression pattern that was not fit in any of the above expression pattern.

### In-gel Digestion

Spots of varied intensities were excised manually and transferred to 1.5 mL microcentrifuge tubes. The protein spots of lower abundance were removed from all the replicate gels, pooled and digested in a single tube. Protein spots were destained twice with 30 mM potassium ferricyanide and 100 mM sodium thiosulfate, and then were rinsed with 25 mM ammonium bicarbonate in 50% acetonitrile. Gel pieces were dehydrated with 100% acetonitrile, dried under vacuum and incubated for 16 h at 37°C with 10 µL of 10 ng/µL trypsin in 25 mM ammonium bicarbonate. The resulting tryptic fragments were eluted by diffusion into 50% v/v acetonitrile and 0.5% v/v trifluoroacetic acid.

### Mass Spectrometry

Protein MS was conducted using AUTOFLEX II TOF-TOF (Bruker Daltonics, Germany). Digested protein samples (70% v/v acetonitrile and 0.5% v/v trifluoroacetic) were spotted on an AnchorChipTM plate (1.0 µL) and recrystallized CHCA matrix (Bruker Daltonics, Germany) dissolved in 0.1% TFA/70% ACN (0.5 µL). External standards from the manufacturer dissolved in the same matrix solution and spotted on the fixed positions labeled on the plate. Each sample spot was desalted with 0.01% TFA, and dried completely. The peptide ions generated by autolysis of trypsin (with m/z 2163.333 and 2273.434) were used as internal standards for calibration. The list of peptide masses from each PMF was saved for database analysis.

### Data Analysis

Monoisotopic peptide masses generated from the PMFs were analyzed with Auto Flexanalysis (Bruker Daltonics, Germany) and were searched using using MASCOT distiller 2.2 software (http://www.matrixscience.com/). Matches to protein sequences from the Viridiplantae taxon in NCBInr or MSDB database were considered acceptable if : 1) A MOWSE score was obtained from MASCOT, which rates scores as significant if they are above the 95% significance threshold (*p*<0.05); 2) At least four different predicted peptide masses matched the observed masses for an identification to be considered valid; 3) The coverage of protein sequences by the matching peptides should be higher than 10%; 4) a peptide mass tolerance of 25 ppm; 5) a parent ion mass tolerance of 0.2 Da [23], [24]. In addition, some proteins successfully identified have substantial discrepancies between the experimental and calculated p*I* and *M*r, which could be caused by numerous factors such as posttranslational modification (PTM), polymeric forms of proteins, proteolytic degradation of proteins, matches to proteins from different organisms, or genomic sequence, which could contain segments that are spliced out of the functional protein. Such protein identifications were deemed acceptable as long as the other statistical criteria were met.

### Western Blot Analysis

Total protein extracts from maize dry/24 h imbibed embryos and 48 h germinating *Arabidopsis* seed. The protein was resolved by SDS-PAGE imprinted on membranes, and incubated with monoclonal actin-antibodies MabGpa (Sigma) that recognize different subsets of actin isovariants. The anti-actin monoclonal antibody covalently binds with appropriate horseradish peroxidase-conjugated secondary antibodies (Sigma). Quantification of actin proteins was conducted using ECL kit (Sigma) and X-ray films with short exposure times. Experiments repeated at least twice, coomassie brilliant blue stained gels demonstrate approximate equal loading of protein samples as loading control in maize. In addition, histone H3 was loading control in *Arabidopsis*.

### Isolation of Total RNA and Reverse Transcription

Total RNA was isolated from 24 h imbibed embryos of maize inbred line Zong3 and using a standard Trizol RNA isolation protocol (Life Technologies, USA), and treated with DNase (Promega Corporation, USA) following the manufacturer’s instructions. The amount and quality of the total RNA was checked through electrophoresis in 1% agarose gel. The concentration of RNA was measured by spectrophotometer NanoDrop, ND1000 (Nano Drop Technologies). Equal amounts of 2 µg total RNA was reverse transcribed to cDNA in 20 µL reaction using M-MLV reverse transcriptase (Promega Corporation, USA). Reverse transcription was performed for 60 min at 37°C with a final denaturation step at 95°C for 5 min. Aliquots of 2 µL of the obtained cDNA was subjected to RT-PCR analysis.

### Generation of *Arabidopsis* Transgenic Plants

The full-length coding sequence of *ZmACT2* (GRMZM2G006765) was cloned into the plant transformation vector pEGAD between *BsrGI* and *EcoRI* sites. After that, a 1992 bp promoter sequence of *Arabidopsis AtACT7* gene was ligated into the *pEGAD-ZmACT2* between *PacI* and *AgeI* sites, in which *ZmACT2* was drived by *AtACT7P* promoter. The primer sequences were as follows: ZmACT2F: 5′-ACGAGCTGTACAATGGCTGACGGCGAGGACAT-3′, ZmACT2R: 5′-GGAAT -TCTTAGAAGCACTTCCGATGAA-3′ and AtACT7PF: 5′-CCTTAATTAAGATTAT -TTAAGTTGCCAACCAAGC-3′, AtACT7PR: 5′-AGCGCTACCGGTCACTAAAA -AAAAAGTAAAATGAAACCG-3′. Plasmid *AtACT7P::ZmACT2* was introduced into the Agrobacterium tumefaciens strain *GV3101* and used to transform *Atact7* (*Salk_131610*) *Arabidopsis* mutant plants using a floral dip method. Transgenic plants were selected by herbicide, and two representative T3 homozygous lines were obtained for seed germination analysis. Seeds were sterilized and plated on GM medium containing 0.9% agarose but no sucrose. After 3 days of stratification at 4°C in the dark, plates were incubated at 20°C in the darkness. We recorded the germination rate at 12, 24, 36, 48 and 60 h after germination. Experiments were done in triplicate with 70 seeds for each experiment and genotype. Statistical analyses of the differences in germination rate were performed using *t*-test.

## Results

### Heterosis of Radicle Emergence in Maize Hybrid Zong3/87-1

**Figure 1:**
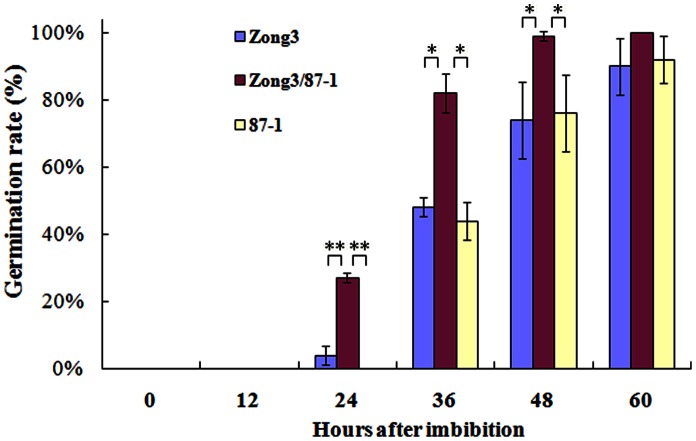
Seed germination rate of maize hybrid Zong3/87-1 and its parents. HAI, hours after imbibitions *, represent Significance level *p*<0.05; ** represent Significance level *p*<0.01.

Growth patterns of germinating seeds in a maize hybrid and its parental lines were shown in Figure 1 and Table S1. It can be noted that, after 24 h of imbibitions at 28°C, 27% of hybrid seeds showed visible radicle emergence, but the percentage of germinated seeds for parental lines Zong3 and 87-1 were only 4% and 0%, respectively. This superiority of hybrid Zong3/87-1 was maintained throughout 48 h when all the seeds were fully germinated, indicating that hybrid Zong3/87-1 exhibited an earlier onset or heterosis in radicle emergence.

Previous study indicated that small kernel weight exhibited a faster and a more efficient germination in maize [25]. In this study, one hundred dry grains weight (HGW) per genotype were record also. The HGW of hybrid Zong3/87-1 (32.15±0.41 g) did not show any significant difference with respect to its female parent Zong3 (32.05±0.36 g), but lower than that of male parent 87-1 (36.93±0.24 g) (*p*<0.05). Therefore, it can be concluded that kernel weight was not related to our observed heterosis in terms of germination rates.

### Two-Dimensional Electrophoresis Analysis

In present study, dry and 24 h imbibed seed embryos of maize hybrid and its parental lines were used for high-throughput two-dimensional gel electrophoresis analysis. In total, 1140 and 1443 protein spots were reproducibly detected in dry and 24 h imbibed seed embryos of the three genotypes, respectively, among which 134 (11.75%) and 191 (13.24%) spots were found to be differentially expressed between hybrids and its parental lines (Table 1, Figure S1).

**Table 1:** The differentially expressed protein spots of additive and nonadditive between maize hybrid and its parental lines.

Stagesa)	No. of displayed protein spots	Differentially expressed pattern									
		Additive				Nonadditive					Total
				++b)	+c)	+/−d)	De)	−f)	−g)	Sum	
DS	1140	71		6	12	2	1	8	34	63	134
24 HAI	1443	125		18	18	4	5	9	12	66	191

a) DS, dry seed; 24 HAI, 24 hours after imbibitions;

b) ++: above high parent expression;

c) +: high parent expression;

d) +/−: partial dominance expression;

e) D: different from additivity (midparent value), not belonging to any of the other classes;

f) −: low parent expression;

g) −−: Below low parent expression.

**Figure 2:**
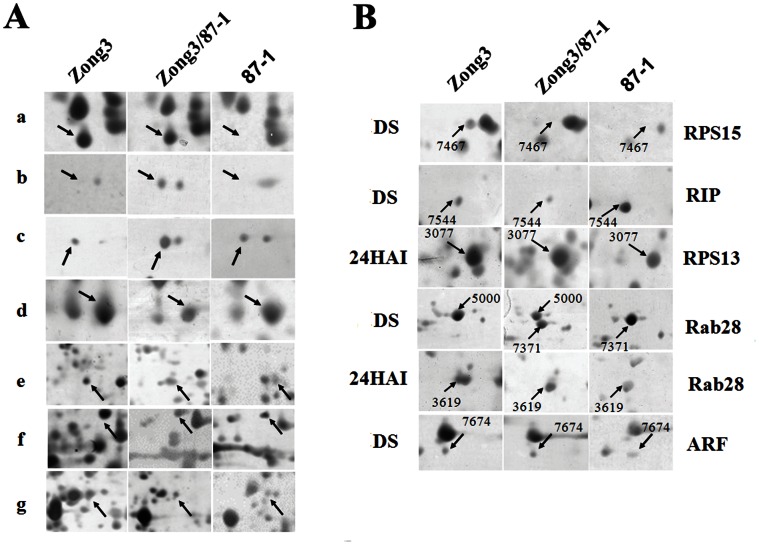
Differential protein expression patterns and some some identified protein spots between hybrids and its parents. A Differential protein expression patterns between hybrids and its parents in maize seed embryos. a) additive expression (spot 7262); b)++: above high parent expression (spot 7228); c)+: high parent expression (spot 7554); d)–: below low parent expression (spot 6686); e) –: low parent expression (spot 2992); f) +/−: partial dominance expression (spot 6547); g)D: different from additivity (midparent value), not belonging to any of the other classes (spot 2728); B. Differential expression patterns of some identified protein spots between hybrids and its parents. DS, dry seed; 24 HAI, 24 hours after imbibitions.

When comparing the patterns of differentially expressed protein spots between hybrid and midparent value (the average value of the parental inbred lines), it was found that a large proportion of proteins in dry embryos (71/134, 52.99%) and 24 h imbibed embryos (125/191, 65.45%) exhibited expression patterns that are not statistically distinguishable from additivity. Remarkably, although the number of nonaddtive expressed protein spots between hybrid and its parental lines are very close in dry and 24 h imbibed embryos (63 and 66, respectively), significant differences were observed in patterns of nonadditively expressed proteins (Table 1). For example, the number of protein spots expressed above or equal to the level of the higher parent (++ and +) in 24 h imbibed embryos (36) was much higher than in dry embryos (18). On the contrary, 42 protein spots were found to be expressed in hybrid below or equal to the level of the lower parent proteins (– and –), which was twice more than in 24 h imbibed embryos (Table 1, Figure 2A).

For each genotype, comparison of dry and 24 h imbibed embryos 2-DE maps was also performed. It can be seen that the many proteins are differentially expressed between dry and 24 h imbibed embryos, and the number of differentially expressed protens in hybrid, Zong3 and 87-1 were 124, 115 and 110, respectively. Further analysis revealed that 68 protein spots existed in 24 h imbibed embryos of hybrid, but they could not be detected in dry embryos of hybrid, which was much higher than that of its two parental lines (40 and 40) (Table S2).

### Identification of Differentially Expressed Protein Spots

All differentially expressed protein spots between maize hybrid and its parental lines were eluted from representative 2-DE gels for identification and 155 protein spots were successfully identified, among which 69 and 86 were derived from dry and 24 h imbibed embryos, respectively (Table S3 and S4). Differential expression patterns of some identified protein spots between hybrids and its parents were shown in Figure 2A. According to the criteria used previously [26], these identified differentially expressed protein spots were classified into eight functional classes, including transcription & translation, energy & metabolism, signal transduction, disease & defense, storage protein, transposable element, cell growth & division, and unclassified proteins (Figure S2). Except for a high portion of unclassified proteins, the largest category in dry and 24 h imbibed embryos is transcription & translation (22% and 30%), followed by energy & metabolism (16% and 19%), disease & defense (15% and 13%) and storage protein (10% and 17%). It was to be noted that a large proportion (9%) of the identified differentially expressed proteins in dry embryos of hybrid and its parental inbred lines was involved in signal transductin, whereas low proportion (2%) proteins in this category was identified in 24 h imbibed embryos.

Further analysis revealed that 155 protein spots identified in this study derived from 118 different genes or gene families, among which 20 proteins representing 39 protein isoforms were found in multiple spots. Additive expressed protein spots identified as protein disulfide isomerase (spot 2174, 2226, 2227, 3257, 6544 and 6562) and Rab28 (spot 5000 and 7371) were only expressed in one parent and hybrid, indicating dominant inheritance from Zong3 and 87-1, which might be a complementation in the hybrid. Some proteins exhibited nonadditive expression patterns, such as UDP-glucose pyrophosphorylase (spots 6675 and 6686). Moreover, globulin2 storage proteins (spots 2551, 3239, 3470, 3473, 7288, 7507, 7510 and 7533) cover two different expression patterns (additive and nonadditive), which made them most complicated. These protein spots could represent different modifications of the same gene product. Alternatively, they might also represent closely related members of gene families, which could not unambiguously identified. Strikingly, there were 17 differentially expressed protein spots were overlapped in dry and 24 h imbibed embryos, among which 4 protein spots displayed the same expression patterns (Table 2).

**Table 2:** Differentially expressed proteins overlapped in dry and 24 h imbibed embryos.

Spot Number (DS/24 HAI)a)	Differential Expression Patternb) (DS/24 HAI)	Functions	Ac. Number
7543/3462	+/D	Predicted protein	gi|168056539
7544/3453	−/additive	Ribosome-inactivating protein	gi|18149181
7551/3479	additive/additive	Putative polyprotein	gi|57863895
7529/3460	+/additive	Vicilin-like embryo storage protein	gi|22284
7228/3008	++/−	Unnamed protein product	gi|116058145
7163/3093	additive/++	Histone deacetylase superfamily (ISS)	gi|116055532
7458/3412	additive/+	Glutathione transferase30	gi|162458953
7510/3470	+/additive	Globulin 2	gi|228310
7455/3405	+/additive	Hypothetical protein At1g03160	Q67Z21_ARATH
7421/3448	+/additive	FZL	Q1KPV0_ARATH
7234/3179	additive/additive	Cyclo-DOPA 5-O-glucosyltransferase	Q59J81_MIRJA
7110/2992	additive/−	Hypothetical protein CHLREDRAFT_118990	gi|159474678
6674/2614	−/additive	ATPase subunit 1	gi|94502565
7377/3257	additive/additive	Protein disulfide isomerase	gi|145666464
7473/3423	additive/−	Hypothetical protein CHLREDRAFT_167615	gi|159485762
7278/3223	additive/additive	Cinful1 polyprotein	Q7XBD4_MAIZE
7533/3546	+/additive	Globulin 2	gi|228310

a) : DS, dry seed; 24 HAI, 24 hours after imbibitions.

b) : ++: above high parent expression; +: high parent expression; D: different from additivity (midparent value), not belonging to any of the other classes; –: low parent expression; –: below low parent expression.

### Complementation of *Arabidopsis Atact7* Allele with Maize Hybrid Upregulated Gene *ZmACT2*

**Figure 3:**
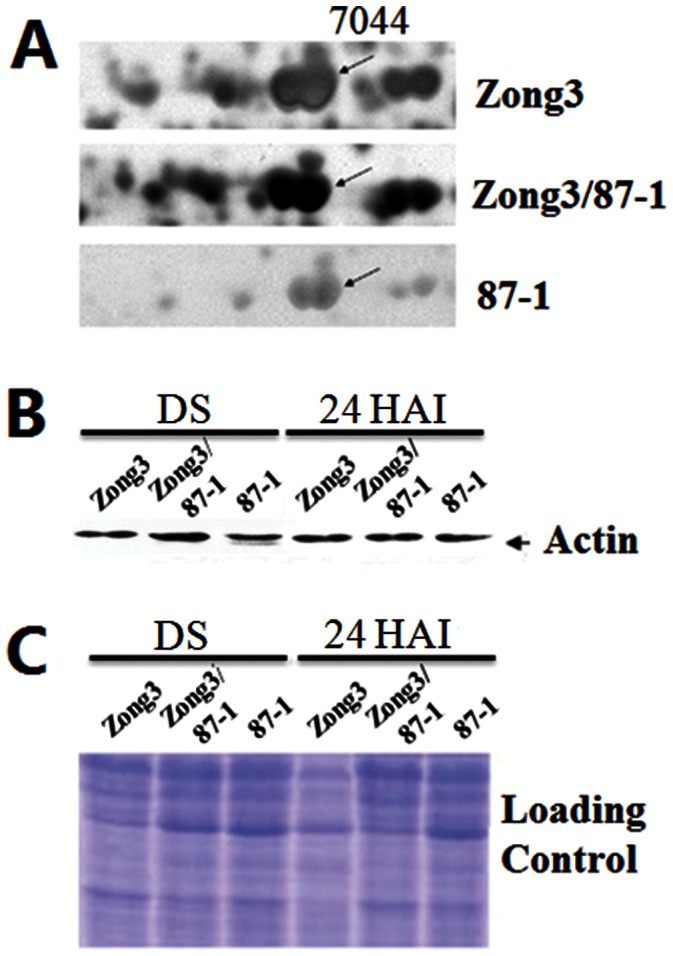
Analysis of actin expression during germination of maize dry and 24 h imbibed seeds embryos at the protein level shown by western immunoblotting with monoclonal antibodies MabGpa. A, The presence of actin isoforms in the maize embryo was analysed by 2-DE. B, Western immunoblotting detected the 42 kDa actin. Equivalent amounts of protein (20 µg) were loaded. C, Protein profile obtained on a 12.5% polyacrylamide gel stained with Coomassie Brilliant Blue. DS, dry seed; 24 HAI, 24 hours after imbibitions.

**Figure 4:**
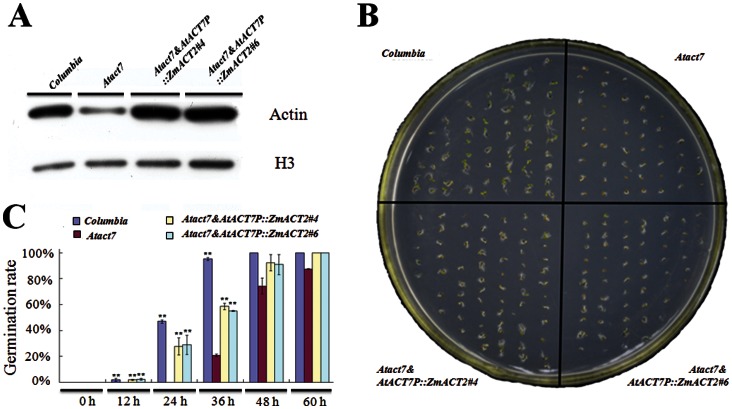
The germination phenotypes of the *Atact7* mutants and complementation of the *Atact7* allele with the maize gene *ZmACT2*. A, Actin protein expression in the *Columbia*, *Atact7* mutant and complementation of the *Atact7* allele with the maize *ZmACT2*. The delay in germination is restored in *Atact7* mutants by the presence of *ZmACT2* as shown for two complemented lines (*Atact7&AtACT7P*::*ZmACT2#4* and *#6*). Total protein extracts from 48 h germinating seeds was resolved by SDS-PAGE and western immunoblotting detected the actin, hisone H3 as loading control. B, Seeds were stratified at 4°C in the dark for 48 h and incubated at 20°C with MS media for seed germination. C, Seed germination of *Arabidopsis thaliana Columbia*, *Atact7* mutants and complementation of the *Atact7* mutant with the maize gene *ZmACT2*. Germination percentage was counted with time course. Asterisks indicate statistically significant differences compared with the *Atact7* mutants. (**Represent Significance level *P*<0.01).

In *Arabidopsis*, it has been reported that *Atact7* mutant display delayed and less efficient in germination [27]. In present study, we also found that one actin protein (designated as ZmACT2 hereafter) was upregulated in dry embryos of maize hybrid (Spot 7044, Figure 3A), which was selected for further analysis. Firstly, western blot analysis was performed on protein samples from dry and 24 h imbibed embryos of maize hybrid Zong3/87-1 and its parental lines. Equal protein loading was confirmed by staining replicate gels with Coomasie Brilliant Blue (Figure 3B and C). Although MAbGPa could recognize all actins, it clearly showed that the abundance of actin protein in dry embryos of maize hybrid was much higher than in its two parental lines, which confirmed the result of 2-DE; Secondly, in order to obtain the full-length cDNA corresponding to hybrid upregulated ZmACT2 protein, RT-PCR was performed with primer pair ZmACT2F/ZmACT2R and open reading frame (ORF) was obtained, which encoded 377 amino acids. Comparison with amino acid sequences in GenBank revealed that the ZmACT2 protein was high similarity (97%) to *Arabidopsis* actin protein 7 (AtACT7) (Data not shown); Finally, to take an insight into the possible function of *ZmACT2* gene in maize, cDNA of *ZmACT2* driven by the promoter of *Arabidopsis AtACT7* gene was transformed into *Atact7* mutant. Western blot analysis indicated that the total actin increased at least one fold in seedling of two T3 homozygous transgenic lines (*AtACT7P::ZmACT2#4* and *6*), as compared to *Atact7* mutant (Figure 4A). As shown in Figure 4B and C, after 36 h imbibitions at 20°C under dark condition, the percentage of germinated seeds for *AtACT7P::ZmACT2* transgenic lines was about 60%, which was lower than that of *Columbia* (80%), while only 20% of *Atact7* seeds showed visible radicle. Collectively, it can be concluded that *ZmACT2* was able to partially rescue the phenotype of the *Atact7* mutant in *Arabidopsis*.

## Discussions

### Significant Alterations in Protein Expression Occurred in Embryos of Hybrids and it Parental Lines During Seed Germination

In most flowering plants, seed germination is the first and may be the foremost growth stage in the plant’s life cycle. Previous studies and experimental data demonstrated that maize hybrid F_1_ seeds have a superior germination capacity in comparison with their parental lines, but the molecular basis for the heterosis in terms of radical emergence is unknown [20]. Recently, a proteomic study on mature embryos of rice hybrid and parents detected 150 differentially expressed protein spots [28]. In maize seed embryos of 24 h imbibitions, 257, 363, 351, 242, and 244 nonadditively expressed proteins were identified in hybrids Zhengdan 958, Nongda 108, Yuyu 22, Xundan 20, and Xundan 18 corresponding to their parents, respectively [21]. In the present study, the number of differentially expressed protein spots between hybrids and its parental lines in dry and 24 h imbibed seed embryos were 134 and 191, respectively, among which 47.01% (63/134) and 34.55% (66/191) protein spots displayed nonadditively expressed pattern. These results indicated that significant alterations in protein level occurred in hybrids as compared to their parents, which might correlate with the observed heterosis.

During the last decades, with the advent of quantitative trait locus (QTL) mapping, three types of QTL interactions were proposed to contribute to heterosis in different studies, i.e., overdominance in maize [29], dominance in rice [30], and epistasis in rice [31]. More recently, however, Hua *et al.* proposed that all kinds of genetic effects, including partial dominance, complete dominance, and overdominance, as well as epistasis contributed to heterosis in a rice “immortalized F_2_” population [32], indicating that these genetic effects were not mutually exclusive in the genetic basis of heterosis. At transcriptional level, all possible modes of gene action, including additivity, high- and low-parent dominance, underdominance, and overdominance, are observed in a global comparison of gene expression in F_1_ hybrid and its inbred parents of maize, rice, and wheat [3], [4], [6], [8]. In this study, 47.01% (63/134) and 34.55% (66/191) differentially expressed proteins were accumulated in a nonadditive fashion in the hybrid compared to the average of their parental inbred lines in dry and 24 h imbibed embryos, respectively. These nonadditively accumulated proteins can be grouped into six differential expression patterns, which were equivalent to overdominance (above high parent), underdominance (below low parent), dominance (high, low parent dominance and partial dominance). These findings are consistent with the hypothesis that multiple molecular models contribute to heterosis [3].

### Expression Patterns between Maize Hybrid and its Parental Lines were Different in Dry and 24 h Imbibed Seed Embryos

Up to date, a number of studies have analyzed heterosis associated gene expression between maize hybrids and their parental inbred lines in different organs and developmental stages [3]–[5]. On the protein level, it was reported that 33% of the proteins detected via 2-DE in the hybrid have been more abundant or newly translated in comparison to the parental lines in maize primary root tip [20]. Recently, in a study of 3.5-day-old primary roots, 49% of the proteins detected in the hybrid accumulated in a nonadditive fashion compared to the average of its parental lines [16]. Similarly, in the reciprocal hybrids and their parental inbred lines 25 and 35 days after pollination, 141 of 597 detected proteins (24%) exhibited nonadditive accumulation in at least one hybrid [17]. In present study, 47.01% and 34.55% differentially expressed proteins were accumulated in a nonadditive fashion in dry and 24 h imbibed hybrid embryos, respectively. Moreover, significant differences were also observed in patterns of nonadditively expressed proteins. For instance, in 24 h imbibed embryos, 36 protein spots displayed above or equal to the level of the higher parent patterns, which is much higher than in dry embryos (18). On the contrary, more protein spots displayed below or equal to the level of the lower parent patterns were observed in dry embryos (42) as compare to that in 24 h imbibed embryos (21). In addition, only 17 of 155 identified protein spots were overlapped in dry and 24 h imbibed embryos, among which 4 displayed same expression patterns. Collectively, our present data provide further evidence for the notion that nonadditive protein accumulation in maize depends on the analyzed developmental stage and plant organ [16].

Seed germination is a complicated physiological process, which starts with the uptake of water by the seed (imbibition) and ends when the embryonic axis starts to elongate and the radicle emerges [33]. Upon imbibition, the quiescent dry seed rapidly resumes metabolic activity, and respiration, enzymatic activity, RNA and protein synthesis are fundamental cellular activities reestablished during germination and are a prerequisite for seedling growth [34]. Based on our present data, it can be seen that 54.55% of nonadditively accumulated proteins in 24 h imbibed embryos displayed above or equal to the level of the higher parent patterns (++ and +). Considering the phenomenon that hybrids germinate earlier than its parental lines, it seems reasonable that more proteins are highly expressed in hybrid embryos during seed germination, but the underlying molecular mechanism responsible for these nonadditively expressed proteins is still an area for further investigated.

### Proteins Involved in Transcription and Translation Overrepresented among Differentially Expressed Proteins

To further get an insight into the possible role of each individual differentially accumulated protein spots in heterosis, 155 protein spots were successfully identified from dry and 24 h imbibed embryos, which can be grouped into eight functional classes. Further analysis revealed that, except for unclassified proteins, the largest category is transcription & translation. Recently, in a study of the close *Arabidopsis* relative *Lepidium sativum*, it was found that both transcription and translation play an important role in seed germination [35]. Moreover, in our present study, it was found that significant alterations in protein expression occurred in dry and 24 h imbibed embryos of hybrid as compared to its parental lines. Thus, it was not surprising that a number of identified nonadditive protein spots were involved in gene transcription and protein translation. Although the precise roles of these identified proteins in heterosis are unknown, some have been demonstrated to be related to seed germination in maize and other plant species.

Firstly, the major plant hormones regulating seed germination in diverse plant species are GA and ABA, which promote and inhibit germination, respectively [36]. Abscisic acid (ABA) responsive protein Rab28 has been shown to be ABA-inducible in embryos. During normal germination, the amount of rab28 proteins decreased after 1 day and became undetectable after 2 days imbibed in water [37]. In the present study, three proteins (spots 3619, 5000 and 7371 (Figure 2B)) were identified as Rab28, especially spot 3619 was only highly expressed in 24 h imbibed embryos of parental line Zong3, but not in F_1_. More recently, it has been shown that auxin also plays a critical role in seed germination, at least in part by mechanisms involving interactions between *ARF10*-dependent auxin and ABA pathways [36]. Interestingly, one auxin response factor protein (spot 7674, Figure 2B) was found to be highly expressed in dry seed embryos of hybrid F_1_. However, at present it would be premature to determine how expression changes in genes involved in hormonal signaling transduction in hybrid might affect heterosis, these expression changes might be important to regulate down-stream gene expression in hybrids that affect heterosis in their turn. Consistent with this hypothesis, a comparative proteomic analysis between imbibed embryos of maize hybrids and their parental lines also exhibited that some differentially expressed proteins were involved with germination-related hormone signal transduction [21].

Secondly, ribosomal proteins are major components of ribosomes, and some appeared to be associated with plant development [38]. For example, knock-out of the plastid ribosomal protein S21 causes impaired photosynthesis and sugar-response during germination and seedling development in *Arabidopsis thaliana* [39]. Ribosome -inactivating proteins (RIPs) are a group of toxic proteins that can irreversibly inactivate ribosome for protein synthesis [40]. Previous studies suggested that early synthesized proteins were essential for germination and were programmed by a conserved polyadenylate-containing mRNA, preserved in dry embryos [41]. Therefore, it was not surprising that three ribosomal related proteins were identified to be differentially accumulated between hybrid and its parental lines. The abundance of one protein, which represented ribosomal proteins S13 (RPS13, Spot 3077, Figure 2B) was equal to that of the highly expressed parent line Zong3 in 24 h imbibed embryos, while ribosome-inactivating protein was detected to be down-regulated in dry embryos of hybrid F_1_. Thus, the change in the expression profiles of these proteins may contribute to the observed vigorous growth of hybrid embryos. It should also be noted that one ribosomal proteins S15 (RPS15, Spot 7467, Figure 2B) were detected to be lowly expressed in dry embryos of hybrid F_1_, the causal reason need further investigation.

### Hybrid Upregluated ZmACT2 Partially Rescue the Delayed Germination Phenotype of the *Atact7* Mutant in *Arabidopsis*

As a major component of the plant cytoskeleton, actins are involved in several basic plant developmental processes including the establishment of cell polarity, cell division plane determination, cell wall deposition, and cell elongation [42], and play an essential role in processes like cytoplasmic streaming, organelle orientation and tip growth certain cells [43]. Most notably, in *Arabidopsis*, *Atact7* mutant displayed delayed and less efficient in germination [27]. In maize, it has been reported that maize actin isoforms expression at these early germination growth stages is a highly regulated event [44]. In present study, we found that the abundance of one protein, which represented ZmACT2 protein, was upregulated in dry embryos of maize hybrid. Further analysis revealed that genes encoding ZmACT2 showed high similarity to *Arabidopsis* AtACT7 proteins. Moreover, transgenic analysis exhibited that maize *ZmACT2* was able to partially rescue the phenotype of the *Atact7* mutant in *Arabidopsis*. Taken together, it can be concluded that the altered pattern of *ZmACT2* gene expression at translational level in the hybrid may be responsible for the observed heterosis. However, the underlying mechanism for the upregulation of ZmACT2 protein in maize hybrid is still an area to be further elucidated.

### Conclusion

In this study, we demonstrated that maize hybrid Zong3/87-1 exhibited an earlier onset or heterosis of radicle emergence. At translational level, 11.75% and 191 13.24% were differentially expressed between hybrid Zong3/87-1 and its parental lines in dry and 24 h imbibed embryos, respectively, among which 47.01% (63/134) and 34.55% (66/191) differentially expressed proteins were accumulated in a nonadditive fashion. Remarkably, significant differences were observed for nonadditively expressed proteins between dry and 24 h imbibed embryos, which provided further evidence for the notion that nonadditive protein accumulation in maize depends on the analyzed developmental stage and plant organ. In addition, a total of 155 differentially expressed proteins were successfully identified, among which some have been demonstrated to be related to seed germination in maize and other plant species, including ABA responsive proteins, ribosomal related proteins and actin protein. Moreover, one of the upregulated proteins in F_1_ hybrids was ZmACT2, a homolog of *Arabidopsis thaliana* ACT7 (AtACT7). Expressing *ZmACT2* driven by the *AtACT7* promoter partially complemented the low germination phenotype in the *Atact7* mutant. These results indicated that hybridization between two parental lines can cause changes in the expression of a variety of proteins, and it can be concluded that the altered pattern of gene expression at translational level in the hybrid may be responsible for the observed heterosis.

## Supporting Information

Figure S1 **Seed embryo 2-DE gel reference maps of maize hybrid Zong3/87-1 and inbred lines Zong3, 87-1 at dry and 24 h after imbibitions.** Dry and 24 HAI indicated that dry and 24 h after imbibed embryo, respectively. Spot number displayed the location of differentially expressed proteins between hybrid Zong3/87-1 and its parental lines in dry and 24 h after imbibitions seed embryo on the 2-DE gel. Protein spots exhibiting changes in intensity are indicated, along with their spot number.
(TIF)

Figure S2 **Functional classification of the identified proteins and protein isoforms differentially expressed in dry and 24 h imbibed seed embryos between hybrid and its parental lines.**
(TIF)

Table S1 **The results of** ***t*** **-test for the germination rate differences between maize hybrid and its parental lines.**
(XLS)

Table S2 **Changes in the number and intensity of protein spots in dry and 24 h imbibed seed embryo.**
(DOC)

Table S3 **The results of** ***t*** **-test for the protein spots accumulation differences between maize embryo hybrid and its parental lines.**
(XLS)

Table S4 **The 155 identified differentially expressed proteins between hybrid and its parental lines.**
(DOC)
