# Supplementary figures and images for: Comparative Proteomic Analysis of Embryos between a Maize Hybrid and Its Parental Lines during Early Stages of Seed Germination
Source: PLoS One. 2013 Jun 11;8(6):e65867. doi: 10.1371/journal.pone.0065867 (PMC3679168; doi:10.1371/journal.pone.0065867)

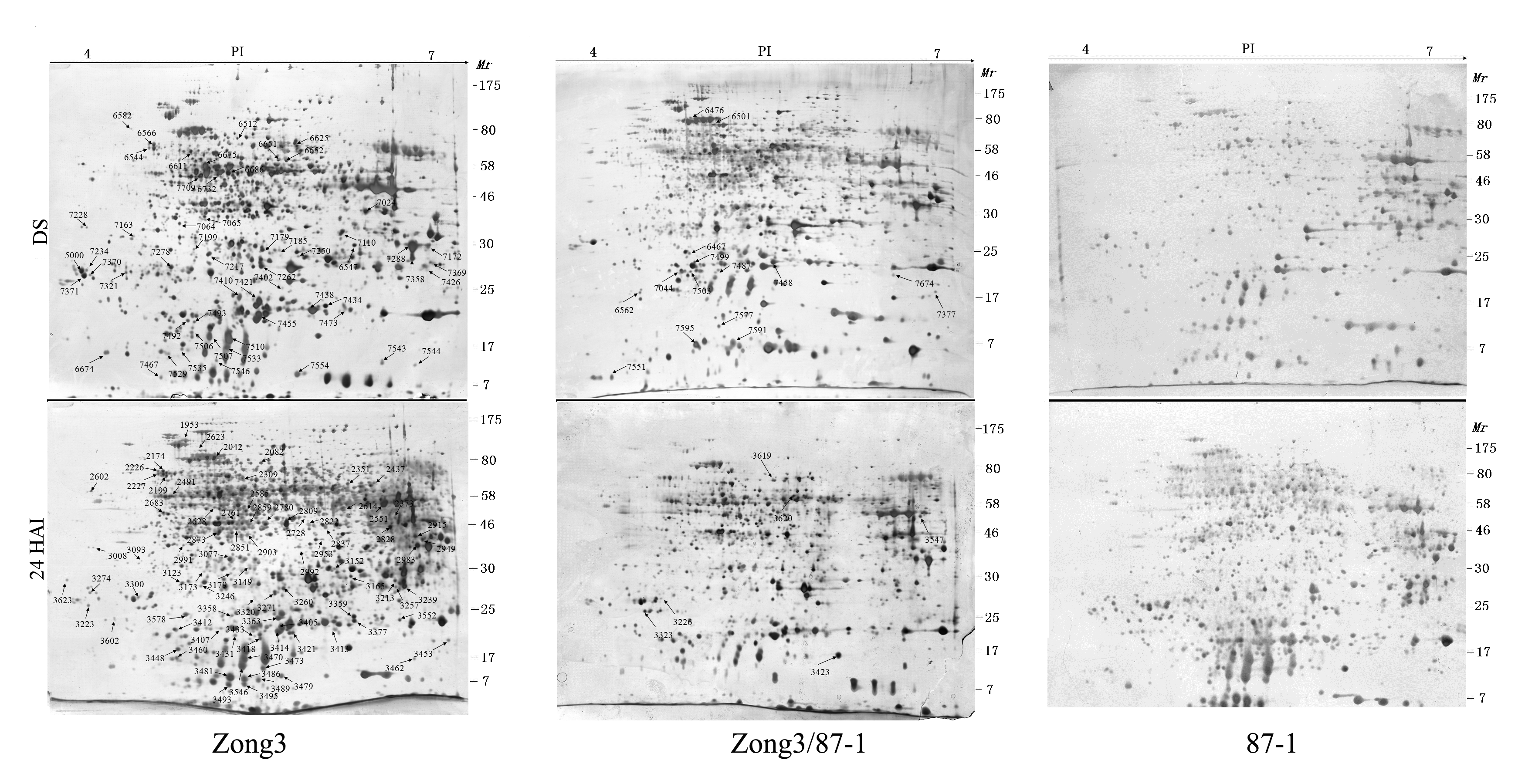

Supplement: Figure S1 — Seed embryo 2-DE gel reference maps of maize hybrid Zong3/87-1 and inbred lines Zong3, 87-1 at dry and 24 h after imbibitions. Dry and 24 HAI indicated that dry and 24 h after imbibed embryo, respectively. Spot number displayed the location of differentially expressed proteins between hybrid Zong3/87-1 and its parental lines in dry and 24 h after imbibitions seed embryo on the 2-DE gel. Protein spots exhibiting changes in intensity are indicated, along with their spot number. (TIF) [file pone.0065867.s001.tif]

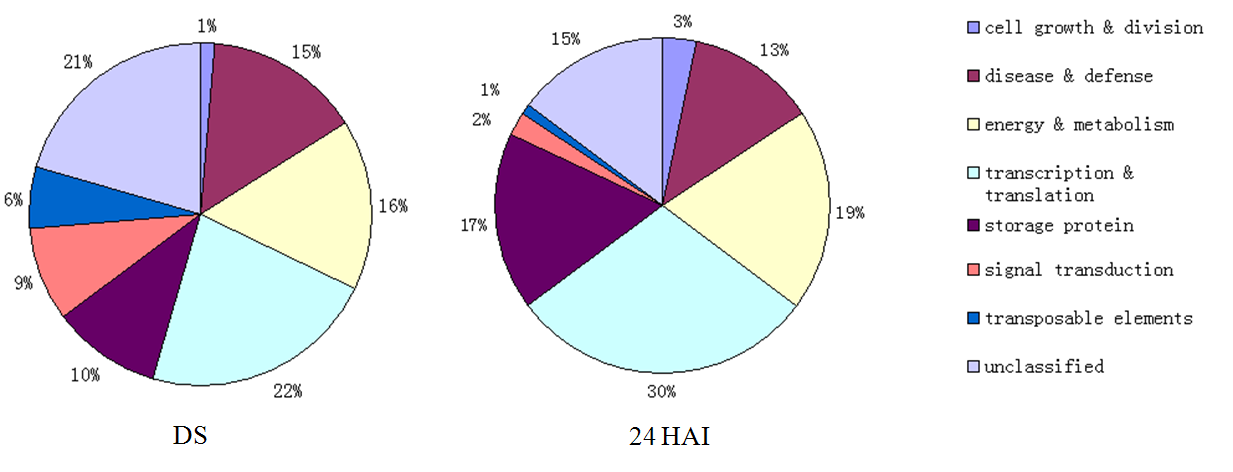

Supplement: Figure S2 — Functional classification of the identified proteins and protein isoforms differentially expressed in dry and 24 h imbibed seed embryos between hybrid and its parental lines. (TIF) [file pone.0065867.s002.tif]
