# Supplementary material for: Comparative Proteomic Analysis of Embryos between a Maize Hybrid and Its Parental Lines during Early Stages of Seed Germination
Source: PLoS One. 2013 Jun 11;8(6):e65867. doi: 10.1371/journal.pone.0065867 (PMC3679168; doi:10.1371/journal.pone.0065867)
Supplement: Table S2 — Changes in the number and intensity of protein spots in dry and 24 h imbibed seed embryo. (DOC) [file pone.0065867.s004.doc]

**Table S2 Changes in the number and intensity of protein spots in dry and 24 h imbibed seed embryo**

| Genotype | A^a)^ | B ^b)^ | C ^c)^ | D ^d)^ | Sum |
| --- | --- | --- | --- | --- | --- |
| Zong3 | 39 | 16 | 40 | 20 | 115 |
| Zong3/87-1 | 20 | 18 | 68 | 18 | 124 |
| 87-1 | 30 | 12 | 40 | 28 | 110 |

a) Number of spots that specifically expressed at dry seed;

b) Number of spots with decreased level from dry to 24 imbibed seed embryo;

c) Number of spots that specifically expressed at 24 h;

d) Number of spots with increased level from dry to 24 imbibed seed embryo;
