# Supplementary material for: Comparative Proteomic Analysis of Embryos between a Maize Hybrid and Its Parental Lines during Early Stages of Seed Germination
Source: PLoS One. 2013 Jun 11;8(6):e65867. doi: 10.1371/journal.pone.0065867 (PMC3679168; doi:10.1371/journal.pone.0065867)
Supplement: Table S4 — The 155 identified differentially expressed proteins between hybrid and its parental lines. (DOC) [file pone.0065867.s006.doc]

**Table S4 The 155 identified differentially expressed proteins between hybrid and its parents**

| **Stages^a)^** | **Spot Number** | **Differential Expression Pattern^b)^** | **Functions** | **Functional categories** | **Taxonomy** | **Ac. number** | **Database** | **Top Score** | **Peptide**  **matched** | **Sequence coverage (%)** |
| --- | --- | --- | --- | --- | --- | --- | --- | --- | --- | --- |
| 24 HAI | 2042 | additive | HSP70 | Disease & defense | *O. sativa* | gi|115452237 | NCBI | 84 | 23 | 28 |
| 24 HAI | 2082 | -- | Unknown | Energy & metabolism | *Z. mays* | gi|194708072 | NCBIr | 70 | 15 | 22 |
| 24 HAI | 2174 | additive | Protein disulfide isomerase | Transcription & translation | *Z. mays* | gi|145666464 | NCBInr | 151 | 23 | 42 |
| 24 HAI | 2199 | +/- | Protein disulfide isomerase | Transcription & translation | *Z. mays* | gi|145666464 | NCBIr | 179 | 27 | 42 |
| 24 HAI | 2226 | additive | Protein disulfide isomerase | Transcription & translation | *Z. mays* | gi|145666464 | NCBInr | 80 | 14 | 26 |
| 24 HAI | 2227 | additive | Protein disulfide isomerase | Transcription & translation | *Z. mays* | gi|145666464 | NCBI | 193 | 27 | 49 |
| 24 HAI | 2309 | additive | Vicilin-like storage protein  Glb1-S, Embryo | Storage protein | *Z. mays* | S21825 | MSDB | 66 | 16 | 26 |
| 24 HAI | 2351 | additive | Aldehyde dehydrogenase | Energy & metabolism | *Z. mays* | gi|195622136 | NCBIr | 85 | 10 | 25 |
| 24 HAI | 2373 | additive | T-CMS restorer factor 2 | Energy & metabolism | *Z. mays* | gi|14276718 | NCBI | 85 | 22 | 31 |
| 24 HAI | 2437 | + | Predicted protein | Unclassified | [*Micromonas sp.*](http://www.ncbi.nlm.nih.gov/Taxonomy/Browser/wwwtax.cgi?lvl=0&id=296587) | gi|255081494 | NCBIr | 77 | 10 | 13 |
| 24 HAI | 2491 | + | Tubulin beta-2 chain | Cell growth & division | *Z. mays* | gi|162463097 | NCBIr | 78 | 10 | 20 |
| 24 HAI | 2551 | additive | Globulin 2 | Storage protein | *Z. mays* | gi|228310 | NCBI | 88 | 22 | 36 |
| 24 HAI | 2585 | + | Aminoacylase-1 | Energy & metabolism | *Z. mays* | gi|226502911 | NCBIr | 204 | 25 | 60 |
| 24 HAI | 2628 | additive | Hexokinase-1 | Energy & metabolism | *Z. mays* | gi|226500740 | NCBIr | 70 | 14 | 34 |
| 24 HAI | 2683 | + | Hypothetical protein | Energy & metabolism | *Z. mays* | gi|226503003 | NCBIr | 68 | 9 | 22 |
| 24 HAI | 2728 | D | Putative elongation factor | Transcription & translation | *A.thaliana* | gi|29824421 | NCBIr | 74 | 20 | 28 |
| 24 HAI | 2761 | additive | Elongation factor Tu | Transcription & translation | *Z. mays* | gi|223949895 | NCBIr | 70 | 13 | 42 |
| 24 HAI | 2809 | additive | Cytosolic 3-phosphoglycerate kinase | Energy & metabolism | *Z. mays* | gi|28172915 | NCBI | 102 | 12 | 52 |
| 24 HAI | 2822 | additive | Unknown | Disease & defense | *Z. mays* | gi|224030793 | NCBIr | 81 | 12 | 37 |
| 24 HAI | 2828 | - | Hairpin-induced family protein | Disease & defense | *I. nil* | gi|97974175 | NCBI | 70 | 7 | 36 |
| 24 HAI | 2837 | additive | Molecular chaperone Hsp90-1 | Disease & defense | *S. lycopersicum* | gi|38154489 | NCBIr | 75 | 13 | 19 |
| 24 HAI | 2851 | additive | Activator of 90 kDa heat shock protein ATPase | Disease & defense | *Z. mays* | gi|226493582 | NCBIr | 137 | 24 | 61 |
| 24 HAI | 2859 | additive | Trehalose synthase-like protein | Energy & metabolism | *G. kirkii* | gi|188509954 | NCBIr | 75 | 10 | 17 |
| 24 HAI | 2870 | additive | Predicted: similar to thioredoxin  -related protein isoform 1 | Transcription & translation | *V. vinifera* | gi|225440207 | NCBIr | 73 | 7 | 22 |
| 24 HAI | 2873 | additive | Glutamine synthetase 5 | Energy & metabolism | *Z. mays* | gi|162459551 | NCBI | 74 | 7 | 18 |
| 24 HAI | 2903 | additive | Putative sorting nexin 1. | Transcription & translation | [*O. sativa*](http://www.ncbi.nlm.nih.gov/Taxonomy/Browser/wwwtax.cgi?lvl=0&id=39947) | Q5N7G9_ORYSA | NCBIr | 71 | 16 | 35 |
| 24 HAI | 2915 | additive | Hypothetical protein | Energy & metabolism | *O. sativa* | gi|125584150 | NCBI | 70 | 9 | 25 |
| 24 HAI | 2949 | - | Predicted protein | Transcription & translation | *O. Lucimarinus* | gi|145347856 | NCBI | 68 | 10 | 12 |
| 24 HAI | 2953 | additive | Small heat shock protein  Hsp23.6 precursor | Disease & defense | *T. aestivum .* | Q9ZP24_WHEAT | NCBIr | 66 | 6 | 33 |
| 24 HAI | 2983 | additive | 20S proteasome, A and B subunits | Transcription & translation | *M. truncatula* | Q2HWA9_MEDTR | NCBIr | 65 | 10 | 26 |
| 24 HAI | 2991 | additive | Hypothetical protein (Fragment). | Signal transduction | *R. idaeus* | Q9SM53_RUBID | NCBIr | 67 | 9 | 28 |
| 24 HAI | 3077 | additive | Ribosomal protein S13 | Transcription & translation | *C. globosum* | gi|22550356 | NCBI | 79 | 10 | 74 |
| 24 HAI | 3123 | additive | Hypothetical protein | Storage protein | *Z. mays* | gi|224030527 | NCBIr | 84 | 20 | 38 |
| 24 HAI | 3149 | + | HOS1 | Transcription & translation | *A.thaliana* | T01011 | MSDB | 72 | 7 | 8 |
| 24 HAI | 3152 | additive | Globulin-1 S allele precursor | Storage protein | *Z. mays* | gi|195658011 | NCBIr | 126 | 14 | 29 |
| 24 HAI | 3165 | ++ | Putative protein | Transcription & translation | *A.thaliana* | gi|4455293 | NCBIr | 71 | 6 | 16 |
| 24 HAI | 3173 | additive | Globulin-1 S allele precursor | Storage protein | *Z. mays* | gi|195658011 | NCBIr | 115 | 17 | 27 |
| 24 HAI | 3213 | + | At5g48610-like protein | Unclassified | *A.thaliana* | Q6XWA2_ARATH | MSDB | 66 | 6 | 31 |
| 24 HAI | 3226 | + | Os02g0602000 | Unclassified | *O. sativa* | gi|115447149 | NCBIr | 73 | 10 | 24 |
| 24 HAI | 3239 | additive | Globulin 2 | Storage protein | *Z. mays* | gi|228310 | NCBI | 101 | 21 | 39 |
| 24 HAI | 3246 | additive | Putative AnthranilatePhosphoribosyltransferase | Energy & metabolism | *O. sativa* | Q6ZG91_ORYSA | MSDB | 68 | 11 | 19 |
| 24 HAI | 3260 | additive | Maturase K | Unclassified | *B. coccinea* | gi|15340912 | NCBIr | 91 | 7 | 27 |
| 24 HAI | 3271 | additive | Os07g0663500 | Energy & metabolism | *O. sativa* | gi|255678042 | NCBIr | 71 | 8 | 17 |
| 24 HAI | 3274 | + | Rab28 | Disease & defense | *Z. mays* | gi|22460 | NCBIr | 89 | 11 | 48 |
| 24 HAI | 3320 | additive | Glycosyltransferase  family 43 protein | Energy & metabolism | *A. thaliana* | Q9SFZ7_ARATH | MSDB | 66 | 9 | 28 |
| 24 HAI | 3323 | additive | Elongation factor 1-delta 1 | Transcription & translation | *Z. mays* | gi|226505926 | NCBIr | 78 | 8 | 31 |
| 24 HAI | 3358 | additive | Putative sorting nexin 1 | Transcription & translation | *O. sativa* | Q5N7G9_ORYSA | MSDB | 78 | 13 | 31 |
| 24 HAI | 3359 | -- | TPA: putative cystatin | Disease & defense | *Z. mays* | gi|71061120 | NCBI | 116 | 12 | 53 |
| 24 HAI | 3363 | additive | Hypothetical protein OsI_037490 | Cell growth & division | *O. sativa* | gi|125537043 | NCBI | 70 | 10 | 24 |
| 24 HAI | 3377 | ++ | Putative kinesin light chain | Energy & metabolism | *A.thaliana* | gi|4432819 | NCBI | 72 | 11 | 15 |
| 24 HAI | 3407 | additive | RNA-binding region RNP-1  (RNA recognition motif) |  | *M. truncatula* | gi|87240850 | NCBIr | 70 | 6 | 28 |
| 24 HAI | 3413 | additive | Hypothetical protein OsI_04526 | Transcription & translation | *O. sativa* | gi|125528465 | NCBIr | 72 | 13 | 30 |
| 24 HAI | 3414 | additive | ATCDT1B/CDT1/CDT1B Cyclin-dependent protein kinase | Cell growth & division | *A.thaliana* | gi|15232570 | NCBI | 69 | 9 | 21 |
| 24 HAI | 3418 | additive | Hypothetical protein | Unclassified | *O. sativa* | Q8W2P8_ORYSA | MSDB | 68 | 9 | 40 |
| 24 HAI | 3421 | additive | Hypothetical protein | Transcription & translation | *C. reinhardtii* | gi|159485518 | NCBI | 69 | 7 | 17 |
| 24 HAI | 3431 | additive | Unknown | Transcription & translation | *P. sitchensis* | gi|116794364 | NCBI | 69 | 7 | 29 |
| 24 HAI | 3433 | additive | Predicted protein | Unclassified. | *P. patens* | gi|168038459 | NCBI | 69 | 7 | 12 |
| 24 HAI | 3473 | - | Globulin 2 | Storage protein | *Z. mays* | gi|228310 | NCBI | 71 | 7 | 17 |
| 24 HAI | 3486 | - | Unknown | Storage protein | *Z. mays* | gi|224030527 | NCBIr | 115 | 12 | 21 |
| 24 HAI | 3489 | additive | Unknown | Storage protein | *Z. mays* | gi|224030527 | NCBIr | 86 | 10 | 19 |
| 24 HAI | 3493 | additive | Unknown | Storage protein | *Z. mays* | gi|224030528 | NCBIr | 96 | 9 | 19 |
| 24 HAI | 3495 | additive | Vicilin-like embryo storage protein | Storage protein | *Z. mays* | gi|22284 | NCBI | 76 | 12 | 18 |
| 24 HAI | 3547 | ++ | Hypothetical protein | Unclassified | *O. sativa* | gi|28812113 | NCBIr | 92 | 8 | 96 |
| 24 HAI | 3552 | additive | DGCR14 protein | Unclassified | *Z. mays* | gi|194699912 | NCBIr | 71 | 10 | 35 |
| 24 HAI | 3578 | + | Atnudt3 | Unclassified | *A.thaliana* | AAM53332 | MSDB | 68 | 7 | 17 |
| 24 HAI | 3602 | additive | Predicted: similar to thioredoxin -related protein isoform 2 | Transcription & translation | [*V. vinifera*](http://www.ncbi.nlm.nih.gov/Taxonomy/Browser/wwwtax.cgi?lvl=0&id=29760) | gi|225440205 | NCBIr | 73 | 6 | 25 |
| 24 HAI | 3619 | -- | Rab28 | Disease & defense | *Z. mays* | gi|22460 | NCBI | 109 | 10 | 50 |
| 24 HAI | 3620 | -- | Hypothetical protein | Unclassified | *O. sativa* | Q654J0_ORYSA | MSDB | 70 | 7 | 24 |
| 24 HAI | 3623 | additive | Hypothetical protein | Disease & defense | *Z. mays* | gi|226530579 | NCBIr | 119 | 30 | 35 |
| 24 HAI | 2614 | additive | ATPase subunit 1 | Energy & metabolism | *Z. mays* | gi|94502565 | NCBIr | 195 | 30 | 51 |
| 24 HAI | 2992 | - | Hypothetical protein | Transcription & translation | *C. reinhardtii* | gi|159474678 | NCBInr | 71 | 9 | 21 |
| 24 HAI | 3008 | -- | Unnamed protein product | Unclassified | *O. tauri* | gi|116058145 | NCBIr | 73 | 11 | 29 |
| 24 HAI | 3093 | ++ | Histone deacetylase  superfamily (ISS) | Signal transduction | *O. tauri* | gi|116055532 | NCBInr | 71 | 16 | 16 |
| 24 HAI | 3179 | additive | Cyclo-DOPA 5-O-glucosyltransferase | Transcription & translation | *M. jalapa* | Q59J81_MIRJA | MSDB | 67 | 7 | 10 |
| 24 HAI | 3223 | additive | Cinful1 polyprotein | transposable elements | *Z. mays* | Q7XBD4_MAIZE | MSDB | 68 | 11 | 15 |
| 24 HAI | 3257 | additive | Protein disulfide isomerase | Transcription & translation | *Z. mays* | gi|145666464 | NCBI | 84 | 8 | 16 |
| 24 HAI | 3405 | additive | Hypothetical protein At1g03160 | Energy & metabolism | *A.thaliana* | Q67Z21_ARATH | MSDB | 67 | 8 | 15 |
| 24 HAI | 3412 | + | Glutathione transferase30 | Energy & metabolism | *Z. mays* | gi|162458953 | NCBI | 99 | 10 | 34 |
| 24 HAI | 3423 | - | Hypothetical protein | Unclassified | *C. reinhardtii* | gi|159485762 | NCBInr | 71 | 11 | 64 |
| 24 HAI | 3448 | additive | FZL. | Signal transduction | *A.thaliana* | Q1KPV0_ARATH | MSDB | 67 | 16 | 23 |
| 24 HAI | 3453 | additive | Ribosome-inactivating protein | Transcription & translation | *S. oleracea* | gi|18149181 | NCBInr | 73 | 13 | 33 |
| 24 HAI | 3460 | additive | Vicilin-like embryo storage protein | Storage protein | *Z. mays* | gi|22284 | NCBInr | 74 | 10 | 19 |
| 24 HAI | 3462 | D | Predicted protein | Transcription & translation | *P. patens* | gi|168056539 | NCBInr | 69 | 12 | 37 |
| 24 HAI | 3470 | additive | Globulin 2 | Storage protein | *Z. mays* | gi|228310 | NCBI | 98 | 13 | 28 |
| 24 HAI | 3479 | additive | Putative polyprotein | Transcription & translation | *O. sativa* | gi|57863895 | NCBI | 70 | 11 | 10 |
| 24 HAI | 3546 | additive | Globulin | Storage protein | *Z. mays* | gi|228310 | NCBInr | 112 | 14 | 30 |
| DS | 5000 | additive | Rab28 | Disease & defense | *Z. mays* | gi|22460 | NCBInr | 98 | 10 | 48 |
| DS | 6467 | -- | Heat shock protein 70 homolog 2 | Disease & defense | *Z. mays* | gi|6016150 | NCBInr | 102 | 18 | 32 |
| DS | 6501 | -- | Heat shock protein 70 | Disease & defense | *C. sativus* | gi|6911553 | NCBInr | 82 | 15 | 27 |
| DS | 6512 | additive | Glucose phosphomutase 1 | Energy & metabolism | *Z. mays* | gi|12585309 | NCBInr | 111 | 22 | 29 |
| DS | 6544 | additive | Protein disulfide isomerase | Transcription & translation | *Z. mays* | gi|145666464 | NCBInr | 166 | 24 | 43 |
| DS | 6547 | +/- | Globulin1 | Storage protein | *Z. mays* | gi|162463479 | NCBInr | 99 | 15 | 35 |
| DS | 6562 | additive | Protein disulfide isomerase | Transcription & translation | *Z. mays* | gi|145666464 | NCBInr | 81 | 13 | 27 |
| DS | 6566 | ++ | Protein disulfide isomerase | Transcription & translation | *Z. mays* | gi|145666464 | NCBInr | 178 | 26 | 43 |
| DS | 6582 | -- | Calcium-binding allergen Ole e 8 | Signal transduction | [*O. europaea*](http://www.ncbi.nlm.nih.gov/Taxonomy/Browser/wwwtax.cgi?lvl=0&id=4146) | gi|14423648 | NCBInr | 76 | 6 | 49 |
| DS | 6611 | additive | Putative chaperonin 60 beta | Transcription & translation | *O. sativa* | Q9LWT6_ORYSA | MSDB | 81 | 16 | 24 |
| DS | 6625 | additive | Os01g0283000 | Energy & metabolism | *O. sativa* | gi|115436008 | NCBInr | 69 | 9 | 13 |
| DS | 6651 | additive | Unknown | Transcription & translation | *A.thaliana* | gi|21593852 | NCBInr | 70 | 8 | 25 |
| DS | 6652 | additive | Ketol-acid reductoisomerase | Energy & metabolism | *A.thaliana* | gi|15231092 | NCBInr | 80 | 13 | 19 |
| DS | 6675 | + | UDP-glucose pyrophosphorylase | Energy & metabolism | *B. oldhamii* | gi|37729658 | NCBInr | 88 | 20 | 37 |
| DS | 6686 | -- | UDP-glucose pyrophosphorylase | Energy & metabolism | *B. oldhamii* | gi|37729658 | NCBInr | 106 | 17 | 34 |
| DS | 6732 | additive | Hypothetical protein | Cell growth & division | *O. sativa* | gi|125556628 | NCBInr | 74 | 23 | 28 |
| DS | 7024 | additive | Putative protein | Unclassified | *A.thaliana* | gi|3036796 | NCBInr | 76 | 12 | 33 |
| DS | 7044 | ++ | Actin | Cell growth & division | [*Z. mays*](http://www.ncbi.nlm.nih.gov/Taxonomy/Browser/wwwtax.cgi?lvl=0&id=4577) | gi|259490134 | NCBInr | 93 | 16 | 45 |
| DS | 7064 | additive | Unknown protein | Unclassified | *A.thaliana* | gi|15229277 | NCBInr | 70 | 13 | 13 |
| DS | 7065 | - | Transposon protein, | transposable elements | *O. sativa* | gi|62734076 | NCBInr | 76 | 20 | 30 |
| DS | 7172 | additive | Hypothetical protein | Signal transduction | *V. vinifera* | gi|147828183 | NCBInr | 71 | 9 | 40 |
| DS | 7179 | - | Cysteine synthase2 | Energy & metabolism | *Z. mays* | gi|162458737 | NCBInr | 90 | 12 | 40 |
| DS | 7185 | +/- | Putative NB-ARC Domain protein | Disease & defense | *O. sativa* | gi|156600014 | NCBInr | 80 | 13 | 57 |
| DS | 7199 | - | EdeA protein | Unclassified | *T. tobacco* | S35949 | MSDB | 67 | 9 | 47 |
| DS | 7217 | -- | Unnamed protein product | Unclassified | *V. vinifera* | gi|157357935 | NCBInr | 72 | 5 | 68 |
| DS | 7250 | additive | Hypothetical protein | Unclassified | *O. sativa* | gi|21327987 | NCBInr | 69 | 8 | 32 |
| DS | 7262 | additive | Unnamed protein product | Unclassified | *V. vinifera* | gi|157359129 | NCBInr | 70 | 4 | 100 |
| DS | 7288 | - | Globulin 2 | Storage protein | [*Z. mays*](http://www.ncbi.nlm.nih.gov/Taxonomy/Browser/wwwtax.cgi?lvl=0&id=4577) | gi|228310 | NCBInr | 122 | 20 | 42 |
| DS | 7321 | additive | 14-3-3-like protein GF14-6 | Signal transduction, | *Z. mays* | gi|1345587 | NCBInr | 116 | 11 | 44 |
| DS | 7358 | + | Formyltetrahydrofolate deformylase | Energy & metabolism | *B. oleracea* | gi|89257634 | NCBInr | 72 | 10 | 40 |
| DS | 7369 | additive | Predicted protein | Unclassified | *P. patens* | gi|162693247 | NCBInr | 76 | 7 | 35 |
| DS | 7371 | additive | Rab28 | Disease & defense | *Z. mays* | gi|22460 | NCBInr | 125 | 12 | 48 |
| DS | 7402 | additive | NBS/LRR disease resistance-like | Disease & defense | *M. acuminata* | Q8LJV4_MUSAC | MSDB | 67 | 4 | 33 |
| DS | 7410 | + | Globulin-1 S allele precursor | Storage protein | *Z. mays* | gi|121205 | NCBInr | 69 | 10 | 23 |
| DS | 7426 | -- | Os11g0628800 | transposable elements | *O. sativa* | gi|115486335 | NCBInr | 68 | 10 | 16 |
| DS | 7434 | -- | HV1LRR1 | Disease & defense | *H. vulgare* | gi|5669778 | NCBInr | 81 | 17 | 22 |
| DS | 7438 | additive | hypothetical protein-like protein | Disease & defense | *M. nodulans* | gi|163699348 | NCBInr | 73 | 6 | 50 |
| DS | 7467 | -- | Ribosomal protein S15 | Transcription & translation | *O. pumila* | gi|139389476 | NCBInr | 70 | 6 | 46 |
| DS | 7487 | -- | Hypothetical protein | transposable elements | *V. vinifera* | gi|147799576 | NCBInr | 71 | 19 | 21 |
| DS | 7492 | additive | Unknown | Energy & metabolism | *P.sitchensis* | gi|116786848 | NCBInr | 72 | 10 | 25 |
| DS | 7493 | additive | Unknown protein | Transcription & translation | *A.thaliana* | gi|15240560 | NCBInr | 71 | 15 | 15 |
| DS | 7499 | -- | Hypothetical protein | Unclassified | *O. sativa* | gi|27817932 | NCBInr | 74 | 10 | 34 |
| DS | 7503 | -- | Predicted protein | Unclassified | *P. patens* | gi|162667799 | NCBInr | 68 | 12 | 30 |
| DS | 7506 | -- | Hypothetical protein | Unclassified | *V. vinifera* | gi|147820968 | NCBInr | 77 | 23 | 40 |
| DS | 7507 | + | Globulin 2 | Storage protein | *Z. mays* | gi|228310 | NCBInr | 107 | 17 | 34 |
| DS | 7535 | additive | Heat shock protein 17.9 | Disease & defense | *P. glaucum* | gi|1122317 | NCBInr | 91 | 8 | 42 |
| DS | 7546 | additive | Heat shock protein 17.9 | Disease & defense | *P. glaucum* | gi|1122317 | NCBInr | 94 | 11 | 50 |
| DS | 7554 | + | Hypothetical protein | Unclassified | *O. sativa* | gi|47496996 | NCBInr | 69 | 5 | 65 |
| DS | 7577 | additive | S4-RNase protein | Transcription & translation | *P. armeniaca* | Q5MQL6_PRUAR | MSDB | 99 | 6 | 18 |
| DS | 7591 | - | Unknown protein | Energy & metabolism | *A.thaliana* | gi|18410580 | NCBInr | 70 | 8 | 44 |
| DS | 7595 | additive | Hypothetical protein | Unclassified | *O. sativa* | gi|42408378 | NCBInr | 72 | 12 | 25 |
| DS | 7674 | ++ | Auxin response factor-like protein | Signal transduction | *M. indica* | Q84QI6_MANIN | MSDB | 66 | 13 | 19 |
| DS | 6674 | -- | ATPase subunit 1 | Energy & metabolism | *Z. mays* | gi|94502565 | NCBIr | 195 | 30 | 51 |
| DS | 7110 | additive | Hypothetical protein | Transcription & translation | *C.reinhardtii* | gi|159474678 | NCBInr | 71 | 9 | 21 |
| DS | 7163 | additive | Histone deacetylase superfamily (ISS) | Signal transduction | *O. tauri* | gi|116055532 | NCBInr | 71 | 16 | 16 |
| DS | 7228 | ++ | Unnamed protein product | Unclassified | *O. tauri* | gi|116058145 | NCBIr | 73 | 11 | 29 |
| DS | 7234 | additive | Cyclo-DOPA 5-O-glucosyltransferase | Transcription & translation | *M. jalapa* | Q59J81_MIRJA | MSDB | 67 | 7 | 10 |
| DS | 7278 | additive | Cinful1 polyprotein | transposable elements | *Z. mays* | Q7XBD4_MAIZE | MSDB | 66 | 15 | 19 |
| DS | 7377 | additive | Protein disulfide isomerase | Transcription & translation | *Z. mays* | gi|145666464 | NCBI | 84 | 8 | 16 |
| DS | 7421 | + | FZL. | Signal transduction | *A.thaliana* | Q1KPV0_ARATH | MSDB | 67 | 16 | 23 |
| DS | 7455 | + | Hypothetical protein | Energy & metabolism | *A.thaliana* | Q67Z21_ARATH | MSDB | 67 | 8 | 15 |
| DS | 7458 | additive | Glutathione transferase30 | Energy & metabolism | *Z. mays* | gi|162458953 | NCBI | 99 | 10 | 34 |
| DS | 7473 | additive | Hypothetical protein | Unclassified | *C. reinhardtii* | gi|159485762 | NCBInr | 71 | 11 | 64 |
| DS | 7510 | + | Globulin 2 | Storage protein | *Z. mays* | gi|228310 | NCBInr | 71 | 10 | 20 |
| DS | 7529 | + | Vicilin-like embryo  storage protein | Storage protein | *Z. mays* | gi|22284 | NCBInr | 74 | 10 | 19 |
| DS | 7533 | + | Globulin 2 | Storage protein | *Z. mays* | gi|228310 | NCBInr | 112 | 14 | 30 |
| DS | 7543 | + | Predicted protein | Transcription & translation | *P.a patens* | gi|168056539 | NCBInr | 69 | 12 | 37 |
| DS | 7544 | - | Ribosome-inactivating protein | Transcription & translation | *S. oleracea* | gi|18149181 | NCBInr | 73 | 13 | 33 |
| DS | 7551 | additive | Putative polyprotein | Transcription & translation | *O. sativa* | gi|57863895 | NCBI | 70 | 11 | 10 |

a): 24 HAI, 24 hours after imbibitions; DS, dry seed.

b)++: above high parent expression; +: high parent expression; +/-: partial dominance expression; D: different from additivity (midparent value), not belonging to any of the other classes; -: low parent expression; --: below low parent expression.
